# *Agrobacterium tumefaciens*-mediated transformation of corn (*Zea mays* L.) multiple shoots

**DOI:** 10.1080/13102818.2014.907654

**Published:** 2014-07-10

**Authors:** Shi-liang Cao, Pathmalojiny Masilamany, Wen-bin Li, K. Peter Pauls

**Affiliations:** ^a^ Key Laboratory of Soybean Biology in Chinese Education Ministry, College of Agronomy, Northeast Agricultural University, Harbin, Heilongjiang, P.R. China; ^b^ Department of Plant Agriculture, University of Guelph, Guelph, Ontario, Canada

**Keywords:** *agrobacterium tumefaciens*-mediated transformation, corn, plant regeneration, multiple shoots

## Abstract

An *Agrobacterium tumefaciens*-mediated corn transformation method based on multiple shoot tissue cultures was developed, which is effective with a variety of corn inbred lines and standard binary vectors. Six factors that affected the success of corn transformation were tested, including *A. tumefaciens* strain, corn genotype, tissue culture growth stage, medium composition, co-culture temperature and surfactant treatment. Agropine-type bacteria (EHA 101 and AGL 1) were eightfold more effective than octopine-type strain for corn multi-shoot tissues transformation. The average frequency of Glucuronidase (GUS)-positive explants obtained from 14 corn genotypes ranged from 36% to 76%. L-proline (0.7 g L^−1^) in the co-culture medium apparently improved the frequency of transformation. The newly initiated multi-shoot tissues were most responsive to *Agrobacterium* infection. A positive correlation was found between multi-shoot tissue susceptibility to *Agrobacterium* and the proportion of cells in G1 phase. Transformants were identified by reverse transcription Polymerase Chain Reaction (PCR) and by southern blot hybridization assays. The frequency of transformants was approximately 2% based on the number of multi-shoot explants co-cultivated with *Agrobacterium*.

## Introduction

Because of its importance as a food and feed source, corn has been a prime target for genetic manipulation. A number of advances have been made in corn transformation technology since the first production of transgenic corn plants[1] including the reports of the production of fertile transgenic corn plants by physical methods from cell suspension cultures [2,3] and Type-I callus [4] as well as differentiated target tissues such as pre-cultured immature embryos.[5] Fertile transgenic plants have also been produced from immature embryos following *Agrobacterium* co-cultivation.[6,7]

Several agronomically important genes have been incorporated into corn, including the Bt-toxin gene for resistance to European corn borer or Asian borer.[8,9] Improvement of resistance to maize dwarf mosaic virus has been achieved by means of transgenic RNA interference.[10] In addition, corn has been transformed to produce proteases, aprotinin and avidin (Prodigene Inc, US patents 6,087,558; 5,824,870; 5,767,379).

For most of the corn transformations, microprojectile bombardment of Type-II callus, derived from immature embryos, has been used. However, reliance on this system for corn transformation restricts genetic improvement to a few genotypes that are capable of producing somatic embryos, embryogenic callus or cell suspensions from immature embryos. This is a significant limitation since the competence of immature embryos to produce embryogenic callus only occurs in a small number of corn genotypes.[11,12]

In contrast, the corn apical meristem is morphogenically plastic and can be manipulated to produce multi-shoots and somatic embryos in a relatively genotype-independent manner.[13–15] The multi-shoot cultures derived from apical meristems of many corn genotypes can be subcultured indefinitely. Theoretically, the multi-shoot cultures are excellent target tissues for transformation if foreign DNA can be delivered to the meristematic cells. Zhong et al. [16] and Li et al.[17] reported success with corn transformation by bombardment of multi-shoot cultures derived from apical meristems.

Because of the tendency for biolistic procedures to result in multi-insert transgenics,[6] there is interest in utilizing *Agrobacterium tumefaciens* co-cultivation to transfer foreign genes into corn. However, only a few successes of *A. tumfaciens*-mediated transformation in corn have been reported.[6,7,18] This transformation system is still at an early stage of development compared to the methods that have been developed in rice transformation by *A. tumefaciens*.[19–21] In addition, the *Agrobacterium*-based systems described to date for corn rely on the use of embryogenic callus derived from immature embryos,[6,7,22] which restricts the utilization of transformation in corn improvement strategies. Furthermore, the protocols utilize supervirulent strains of *Agrobacterium* developed by Japan Tobacco Inc,[18] which places additional intellectual property constraints on the use.

In this report, we describe the recovery of transgenic corn plants from multiple shoot cultures co-cultivated with *A. tumefaciens* containing a standard T-DNA vector. Eleven North American inbred lines and three Chinese inbred lines were tested for their transient GUS expression with four different *Agrobacterium* strains. The cell cycle and several other parameters affecting the co-cultivation of *Agrobacterium* with corn meristematic tissues were also optimized in this study.

## Materials and methods

### Plant materials and tissue preparation

Fourteen genotypes that had high multi-shoot formation frequencies were selected for the transformation experiments, including 11 genotypes (CG69, CG59, CG65, CG68, CG101, CG94, CG37, CG102, CG103, CG74 and CG93) from Canada [13] and three genotypes (He344, K10 and Longfu746) from China. Mature seeds were sterilized in 70% (v/v) ethanol for 5 min and soaked in 50% commercial bleach (5.5% sodium hypochlorite) with 0.1% Tween 20 for 25 min. The surface-sterilized seeds were washed with sterile water and germinated on MS0 medium consisting of Murashige and Skoog (MS) basal medium,[23] without sucrose, in the dark, at 25 °C for 2–7 d.[13] Coleoptilar nodes without coleoptiles and expanded leaves were removed from the seedlings and cultivated on multi-shoot induction (MSI) medium, consisting of MS basal medium, plus 3 g L^−1^ L-proline, 30 g L^−1^ sucrose, 2 mg L^−1^ Benzylaminopurine (BAP), 0.5–1 mg L^−1^ 2,4-D and 0.7% Phytagar (Gibco Lab, Grand Island, NY, USA) (pH 5.75).[13]

### Co-cultivation conditions

Tissue culture and co-cultivation treatment factors were tested for their effect on the efficiency of T-DNA delivery to shoot apical meristem explants derived from germinated seeds and multi-shoots, including plant genotype, *Agrobacterium* strain, bacterial concentration (optical density or OD), explant type, explant age, co-cultivation medium, co-cultivation temperature, surfactant treatment, supplementation with acetosyringone, vacuum infiltration, sonic treatment and vortex treatment.

### *Agrobacterium tumefaciens* and transformation vectors

**Figure 1.**
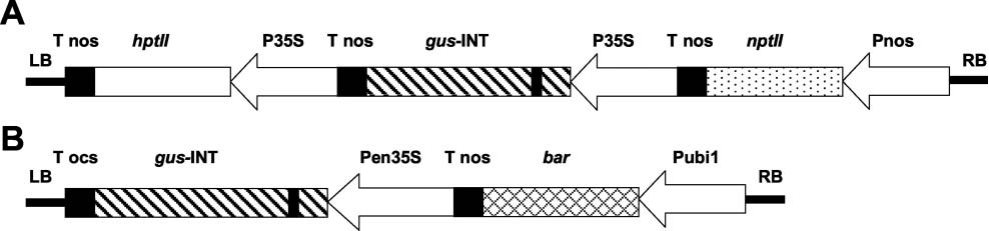
Structures of transformation vectors. (A) pIG121-Hm containing *hpt* II, bacterial hygromycin phosphotransferase gene. p35S: 35S promoter; *gus* -INT: GUS gene plus an intron; *ntp* II: bacterial neomycin phosphotransferase gene; LB: T-DNA left border; RB: T-DNA right border. (B) pBU-35S.IG containing *gus*-INT, GUS gene plus an intron. pen35S: enhanced 35S promoter; *bar*: phosphinothricin acetyl transferase; pubi1: ubiquitin promoter 1.

*A. tumefaciens* strains AGL1,[24] EHA101,[25] C58rifC1 [26] and LBA4404 [27] were used in the transformation experiments. A standard vector pIG121-Hm [28] containing a kanamycin resistance gene (*npt* II), a glucuronidase gene (*gus*A) with an intron in the N-terminal region of the coding sequence [29] and a hygromycin resistance gene (*hpt*II) under the control of the 35S promoter of the cauliflower mosaic virus (Figure 1) was used for some experiments. A standard vector pBU-35S.IG containing a chimeric *Streptomyces hygroscopicus* phosphinothricin (PPT) acetyl transferase (*bar*) gene, a *gus*A*-*catalase intron gene regulated by a ubiquitin 1 promoter (ubi1) and an enhanced 35S promoter was also used (Figure 1). The intron*-gus* gene expresses GUS activity in plant cells but not in *A. tumefaciens* cells. Both plasmids were immobilized into the four *A*. *tumefaciens* strains by triparental mating.[30]

### Co-cultivation, selection and regeneration of transformants

*A. tumefaciens* cultures were grown from −80 °C stocks in Yeast and Peptone (YP) medium (5 g L^−1^ yeast extract, 10 g L^−1^ peptone, 5 g L^−1^ NaCl) supplemented with 50 mg L^−1^ rifampicin and 50 mg L^−1^ hygromycin or 50 mg L^−1^ spectromycin for 48 h at 28 °C. A 48-h *Agrobacterium* culture was diluted to 2 × 10^9^ cfu mL^−1^ with Ainfe liquid medium (ingredients are given below). Freshly isolated shoot tip or multi-shoot cultures were immersed in the bacterial suspension, agitated for 1 min with a vortex mixer (Scientific Industries Inc., NY) and vacuum infiltrated for 30 min. Alternatively, the corn shoot-tips were inoculated by injecting the meristematic regions with the bacterial suspension, using a hypodermic needle (26 1/2 gauge). The inoculated explants were cultivated on solidified Ainfe medium in the dark at 4–30 °C for 4 d. All of the experiments were designed as a complete randomized block with two to three replicates for each treatment and each replicate used 25 explants. After the co-cultivation treatment, the transient GUS expression was evaluated as described below.

To recover putative transgenic plants, the explants were transferred to MSI medium supplemented with 150 mg L^−1^ timentin (SB SmithKline Beecham, Canada) to eliminate *A. tumefaciens*. Two to four weeks later, the tissues were subcultured on MSI medium supplemented with 150 mg L^−1^ timentin and 7 mg L^−1^ hygromycin or 1 mg L^−1^ PPT. After selection for 1–2 months, the survived multi-shoots were subcultured onto rooting medium (MS basal medium, 3% sucrose, 0.7% agar and pH 5.75). The regenerated plantlets were subsequently transferred to Magenta vessels (Sigma, St. Louis, MO, USA) and planted into turface in the growth room.

### Histochemical GUS assay

A modified X-gluc buffer [31] was used, consisting of 100 mmol L^−1^ NaPO_4_ buffer, 10 mmol L^−1^ Na_2_EDTA, 20% methanol, 0.1% Triton X-100 (pH7.0), and 1 mmol L^−1^ 5-bromo-4-chloro-3-indolyl-*6*-D-glucuronic acid cyclohexylammonium salt (X-Gluc) (Diagnostic Chemicals Limited, Charlottetown, Canada). The plant materials were stained in the X-Gluc buffer for 6 h at 37 °C.

### Flow cytometry analysis

Flow cytometry was used to determine the cell cycle distribution in meristems or tissue cultures. Nuclei from meristems of 1–10-d-old seedlings or multi-shoot cultures kept in the dark or at light for 30 d were released by chopping with a razor blade on ice in 1.5 mL nuclei-isolation (NI) buffer as modified from Bino et al. After centrifuging at 7000× *g* for 5 s, the nuclei were resuspended in 300 mL NI buffer with RNAase (1 mg mL^−1^) and were stained by adding 10 mL of propidium iodide (PI) solution.[32] The PI fluorescence of the nuclei was analysed with a Coulter EPICS Elite Cytometer equipped with Argon laser emitting at 488 nm using a 610 nm band pass filter.

### PCR amplification and RT-PCR

Genomic DNA was extracted from leaf tissue with a DNeasy Plant Mini Kit (Qiagen, USA) according to the manufactures instructions. The primers used for GUS gene amplification were: upstream 5′-CCAGACAGAGTGTGATATCTACCCG-3′ and downstream 5′-CATATCCAGCCATGCACACTG-3′. The PCR condition was 94 °C for 3 min and then 94°C for 1 min, 63 °C for 1 min, 72 °C for 2 min with 30 cycles, following 72 °C for 7 min. The expected size of the amplified fragment was 820 bp. The primers used for *hpt*II gene amplification were: upstream 5′-CTCGTGCTTTCAGCTTCGATGT-3′ and downstream 5′-CGAAATTGCCGTCAACCAAGCT-3′. The PCR condition was 94 °C for 3 min and then 94 °C for 1 min, 65 °C for 1 min, 72 °C for 2 min with 30 cycles, following 72 °C for 7 min. The expected size of the amplified fragment was 750 bp. For RT-PCR, total RNA was isolated from leaf tissues with RNeasy kit (Qiagen, Ontario). RNA was reverse transcribed to cDNA with a RETROscript™ kit (Ambion Inc., Austin, TX).

### Southern blot analysis

Genomic DNA was extracted by CTAB (cetyltrimethylammonium bromide) method [33] and 20 μg DNA were digested with *Sma*I *or Eco*RI. The fragments were separated by electrophoresis in 1% agarose gel and blotted onto a nylon membrane (Hybond N, Amersham, England). Pre-hybridization was conducted for 2 h in hybridization solution (Boehringer Mannheim, Indianapolis, IN) at 42 °C. Hybridization was performed at 42 °C overnight in fresh hybridization solution with a 0.82 kb fragment of the GUS coding region labelled with Digoxin (DIG) using PCR DIG Probe Synthesis Kit (Boehringer Mannherim, Indiana, USA). Following hybridization, the membrane was washed twice in 2 × Saline Sodium Citrate (SSC), 0.1% Sodium Dodecyl Sulfate (SDS) for 15 min at room temperature, twice in 0.5 × SSC, 0.1% SDS for 20 min. To detect chemiluminescence, the manufacturer's instructions were followed (BMB, Indianapolis, USA) and X-ray film was exposed to the membrane (XOMAT KODAK) for 15–30 min.

## Results and discussion

### Effects of *Agrobacterium* strains on transient GUS expression

**Figure 2.**
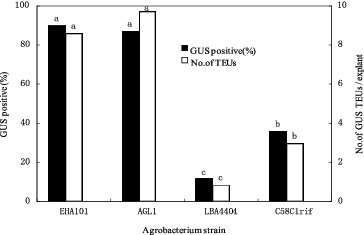
Effectiveness of *A. tumefaciens* strains on inducing transient transgene expression in corn shoot-tip explants 4 d after co-culture. Bars labelled with the same letters are not significantly different (*P* < 0.05) according to Duncan's test (three independent experiments; 25 explants per treatment). Number of TEUs: Number of transient expression units.

Highly regenerable apical tissue was co-cultivated separately with four different *Agrobacterium* strains containing the pIG121-Hm plasmid. Four days after co-cultivation, the interaction between the *Agrobacterium* strains and 14 corn lines was evaluated by testing the tissue for GUS gene expression. The survey showed that large differences existed among the four *A. tumefaciens* strains in their ability to transfer the GUS gene into corn apical tissues. In general, agropine-type *A. tumefaciens* strains (EHA101 and AGL1) were most efficient in inducing transient GUS activity in the apical tissues. On average, 90% and 87% of the explants treated with EHA101 and AGL1, respectively, had GUS-positive cells after 4 d of co-cultivation (Figure 2). The nopaline-type strain (C58rifC1) was less efficient (36%) than the agropine-type strain EHA101, and the octopine-type strain (LBA4404) was approximately one-eighth as effective as EHA101 (Figure 2).

### Effect of corn genotypes on transient GUS expression

**Figure 3.**
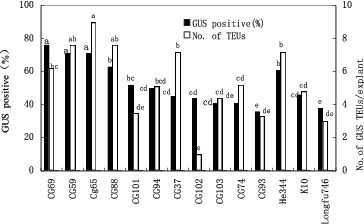
Effects of corn genotypes on transgene expression 4 d after co-culture with different *A. tumefaciens* strains. Means with the same letter are not significantly different at according to Duncan's test.

The corn inbred lines displayed significant differences in their responses to *Agrobacterium* infection (Figure 3). The average frequency of GUS-positive explants across all four *Agrobacterium* strains tested ranged from 36% (CG-93) to 76% (CG-69). The average number of GUS-stained transient expression units (TEUs) in the GUS-positive explants in these experiments ranged from 1 (for CG-102) to 9 (CG-65) for the 14 corn genotypes that were tested (Figure 3).

### Effect of explant age on transient GUS expression

Significant differences were observed among the responses of shoot meristems from seedlings of different ages. The results indicated that apical tissues from the seeds germinated for 4–6 d were highly susceptible to *Agrobacterium*. Apical tissues obtained from younger or older seedlings were not as susceptible to *Agrobacterium* (Table 1).

**Table 1.** Effect of explant age on transformation of corn CG-37 shoot tips and multi-shoot explants co-cultivated with pIG121-Hm/EHA101.

Explant age (d)	GUS-positive ratio (%)	Number of GUS transient expression units; TEUs per explant	Size of GUS
Days after germination (for shoot-tips)			
3	71.9 ± 13.3 c	1.7 ± 1.2 d	NA
4	95.3 ± 2.2 a	9.0 ± 1.6 ab	NA
5	100.0 ± 0.0 a	12.5 ± 2.1 a	NA
6	91.9 ± 7.3 a	12.0 ± 1.3 a	NA
7	88.3 ± 2.4 ab	7.8 ± 2.3 b	NA
8	85.0 ± 7.1 b	4.2 ± 2.0 c	NA
			
Maintenance culture days (for multi-shoots)			
0	98.3 ± 2.4 a	7.1 ± 1.5 a	+++
30	71.7 ± 7.1 a	5.7 ± 1.8 a	+++
60	43.3 ± 9.4 b	2.1 ± 0.2 b	−−−

Note: +++: GUS TEU size > 1 mm; −−−: GUS TEU size < 1 mm; NA: data not available.

Values followed by the same letter were not significantly different at *P* < 0.05 according to Duncan's test.

**Figure 4.**
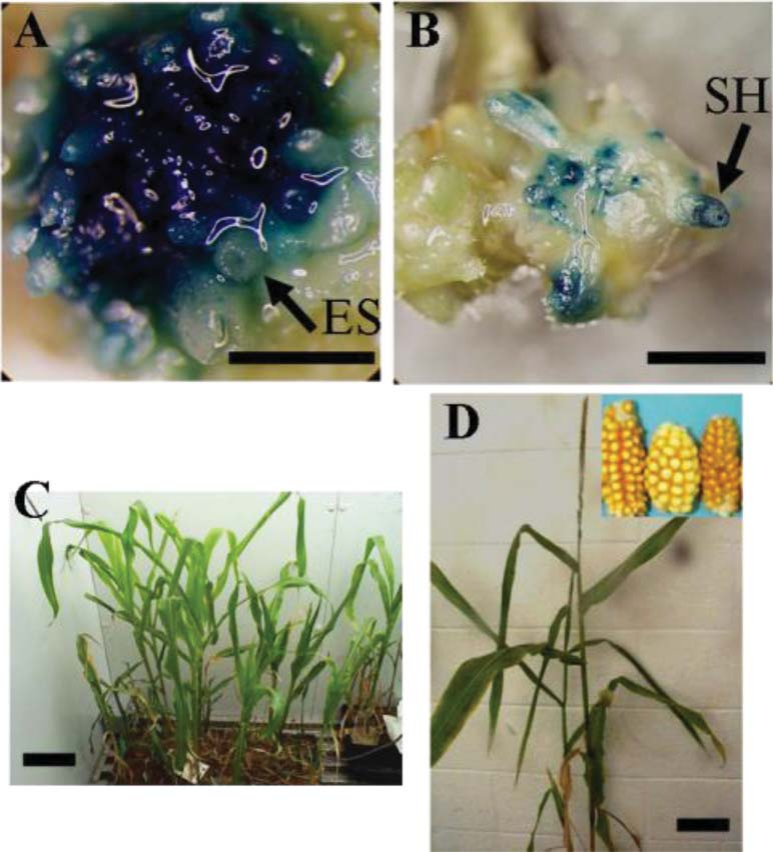
Corn transformation system. Transient GUS activity (A) and stable GUS expression (B) in CG-74 multi-shoot cultures 4 d after co-cultivation with pBU-35S.IG/AGL1. Putative transgenic plants regenerated from CG-65 co-cultivated with pIG121-Hm/AGL1 (C). Confirmed transgenic plant regenerated from CG-65 co-cultivated with pIG121-Hm/AG1 (D). ES: embryogenic structure; SH: shoot initial; bar = 1 mm.

Similarly, the expression of GUS in the multi-shoot cultures obtained from the corn shoot apex explants co-cultivated with *Agrobacterium* was significantly affected by the age of the multi-shoot cultures. The newly produced multi-shoots were most responsive to *Agrobacterium* co-cultivation (Table 1). In these cultures, 98% of the explants stained positive for GUS 4 d after co-cultivation with *Agrobacterium* and these explants had seven GUS-positive TEUs on average. In total, 10%–40% of the explant area was covered by GUS TEUs (Figure 4(A)). However, the susceptibility of the multi-shoot tissue to *Agrobacterium* declined substantially the longer it was subcultured. In particular, the percentage of GUS-positive explants dropped to 43% after 60 d of subculture and the average number of GUS TEUs dropped to two.

### Effects of co-cultivation medium additives on transient GUS expression

Studies of the effects of co-culture media on GUS expression in corn multi-shoot cultures from CG-37 co-cultivated with pIG121-Hm/EHA101 demonstrated that the adding of 0.7 g L^−l^ proline in the medium would improve the frequency of transfer of the GUS gene into the corn tissue, since this treatment had the highest number of GUS-positive TEUs per explant (Table 2). However, a higher level of proline (3 g L^−l^) leads to the over-growth of *Agrobacterium* in the corn tissue, and resulted in a decrease of the average number of GUS TEUs per explant as well as in a decrease of the percentage of the GUS-positive explants.

**Table 2.** Effects of different media on transformation of CG-37 multi-shoots co-cultivated with pIG121-Hm/EHA101.

	Medium component								
Medium	Base medium	Sucrose (%)	L-proline (g L^−1^)	Acetosyringone (mol L^−1^)	2,4-D (mg L^−1^)	BAP (mg L^−1^)	pH	GUS-positive ratio (%)	Number of GUS TEUs per explant
Ainfe1	MS	3	0	200	0.5	2	5.68	100.0 ± 0.0 a	7.2 ± 1.1 b
Ainfe2	MS	3	0.7	200	0.5	2	5.68	100.0 ± 0.0 a	10.9 ± 1.6 a
Ainfe3	MS	3	3	200	0.5	2	5.68	60.5 ± 8.3 b	6.8 ± 1.3 b
Ainfe4	1/2MS	3	0	200	0.5	2	5.68	91.1 ± 5.1 a	7.6 ± 3.2 b

Note: Values followed by the same letter were not significantly different at *P* < 0.05 according to Duncan's test.

### Effects of surfactant on transient GUS expression

**Figure 5.**
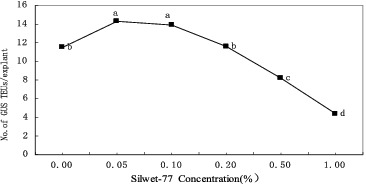
Effect of surfactant Silwet-70 on transient GUS expression in CG-37 multi-shoot culture co-cultivated with pIG121-Hm/EHA101. Means labelled with the same letter are not significantly different according to Duncan's test (three independent experiments; 25 explants per treatment).

To investigate the importance of tissue injury for transformation, the surfactant Silwet-70 was applied in the co-cultivation phase. The results (Figure 5) showed that the addition of 0.05% Silwet-70 significantly promoted the transient GUS expression in apical tissues (*P* < 0.05). At the optimal concentration of Silwet-70 (0.05%), the highest transient GUS expression frequency was 22% higher than the control value. However, higher concentrations of Silwet-70 (>1%) were deleterious to the plant tissues.

### Effect of co-cultivation temperature on transient GUS expression

**Figure 6.**
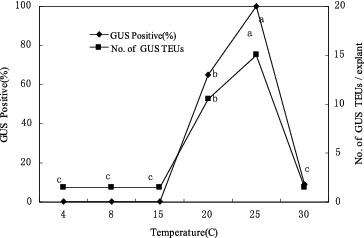
Effect of co-culture temperature on transformation of CG-37 with pIG121-Hm/EHA101. Mean responses at each temperature that are labelled with the same letter are not significantly different at according to Duncan's test (two independent experiments; 25 explants per treatment).

The co-cultivation temperature had a strong effect on the transient GUS expression in the CG-37 shoot-tip cultures co-cultivated with EHA101/pIG121-Hm (Figure 6). At 15 ºC or lower, no GUS activity was observed in apical meristematic tissues and a low frequency of GUS expression was exhibited at 30 ºC. Highly efficient T-DNA delivery (100%) was observed in the cultures co-cultivated at 25 ºC.

### Role of the host cell cycle in the *Agrobacterium*-mediated genetic transformation and the transient GUS expression

**Figure 7.**
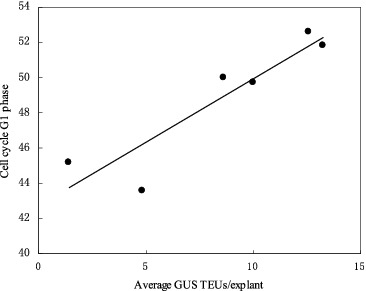
Correlation between the percentage of cells in the tissues in G1 phase of the cell cycle at the time of co-cultivation initiation and the extent of transient GUS expression in shoot tissues. Shoot-tips of CG-37 were co-cultivated with pIG121-Hm/EHA101. GUS TEUs: GUS transient expression units.

Flow cytometric measurements of the proportion of cells in the different stages of the cell cycle in seedling explants (2–7 cm in length) showed that GUS activity in the explants was significantly correlated (*R* ^2^ = 0.86, *P* < 0.05) with the percentage of cells in G1 stage of the cell cycle during the co-culture period (Figure 7).

### Regeneration of putative transgenic plants and detection of gene integration

The optimized *Agrobacterium* co-culture procedure described above was used to produce stable corn transformants. Two to four weeks after co-culture, samples of the multi-shoots were evaluated for GUS activity. These resulted in the recovery of corn tissues that stably expressed the GUS gene (Figure 4(A) and (B)). Explants that were not co-cultivated for the same period did not stain blue with GUS substrate (data not shown). The multi-shoot clumps from co-cultivation treatments were transferred to Murashige and Skoog Modified (MSM) medium containing 150 mg L^−1^ timentin and 1 mg L^−l^ bialaphos or 7 mg L^−l^ hygromycin to select transformed tissues. Two weeks later, the surviving tissues were transferred to MSM medium containing 3 mg L^−l^ bialaphos or 30 mg L^−l^ hygromycin. After approximately six weeks, the clusters of newly regenerated shoots were excised and cultured on shoot elongation medium and then on rooting medium. The rooted shoots were transferred to Magenta vessels. When the plantlets were well established, they were transferred to pots in the growth room for further development (Figure 4(C) and (D)).

**Figure 8.**
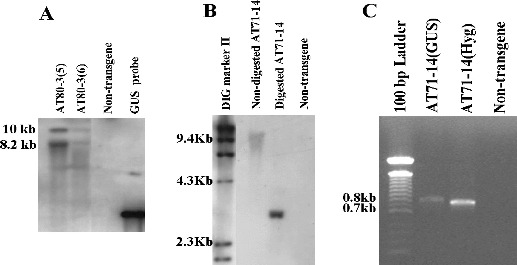
Transgene integration and expression in plants derived from corn multi-shoot co-cultivations with *A. tumefaciens.* (A) Southern blots for plants selected from of CG-65 co-cultivated with pBU-35S.IG/AGL1. Lanes 1 and 2: transgenic plants; Lane 3: non-transgenic control; Lane 4: GUS gene fragment. (B) Southern blot for a plant selected from CG-74 co-cultivated with pIG121-Hm/AGL1. Lane 1: DIG molecular marker; Lane 2: non-digested transgenic DNA; Lane 3: digested transgenic DNA; Lane 4: non-transgenic control. (C) RT-PCR detection using GUS gene primers and hygromycin gene primers and cDNA prepared from leaf tissue of AT71-14. Lane 1: 100 bp DNA ladder; Lane 2: cDNA from transgenic CG-74 (AT71-14) and amplified by GUS primers; Lane 3: cDNA transgenic CG-74 (AJ71-14) amplified with hygromycin primers.

To identify putative transformants, PCR analysis was performed separately with GUS gene primers and hygromycin gene primers. From 288 R_0_ plants that were analysed, 12 plants were PCR-positive (data not shown). The putative transformants were further analysed for the integration of GUS into the plant genome, using southern hybridization. A *Sma* I-digested or *EcoR* I-digested genomic DNA from the R_0_ plants yielded 8.5 and 10 kb fragments in transformants AT80-3 [2] and AT80-3 [28] and a 3 kb fragment in the putative transformant AT71-14, respectively (Figure 8(A) and (B)). Southern blot analysis revealed that corn transformants contained one or two copies of the transgene. Furthermore, RT-PCR of cDNA from AT71-14 tissue produced an amplification product with the GUS gene and hygromycin gene primers (Figure 8(C)), indicating that both genes are expressed. The frequency of stable transformants was approximately 2% based on the original number of multi-shoot explants that were co-cultivated.

### Final remarks

We have described an *A. tumefaciens*-mediated transformation protocol for corn that is applicable to a range of corn genotypes that have the ability to produce multi-shoot cultures from seedling shoot-tip explants. Some fundamental problems that transformation methods for cereals share include the loss of embryogenic capacity during long-term culture of protoplast or suspension cultures, genotype dependence and relatively high input of labour and energy. The new procedure has several advantages relative to protocols that use immature embryos as a target for transformation. It is very easy to adjust the scale of the experiments to suite these constraints or opportunities because the explant is obtained from a seedling that eliminates the need to maintain donor plants for the production of immature embryos. The relatively genotype-independent nature of multi-shoot production from corn seedling makes the current transformation procedure suitable for a large range of genotypes.[13,34] These features are essential for developing an efficient transformation protocol. With this transformation method, we could get transgenic plants within 12 weeks.

Southern blot analyses revealed that corn transformants that were produced with this system contained one or two copies of the transgene, similar with the results of Ishida et al. [18] and Vega et al. [7] .The frequency of transformant production based on the number of multi-shoot explants was approximately 2%. Ishida et al. [18] reported 5%–10% transformation frequency with co-cultivated immature embryos with a supervirulent vector. Frame et al. [6] obtained 5.5% transformants from the co-cultivated immature embryos with a standard binary vector. Obviously, our frequency of transformation was lower than the previous reports, which was caused by the elementary difference of the two kinds of plant regeneration systems.

We have reported a significant correlation between the competency of cells in the cultures to regenerate shoots and the proportion of cell in the G1 phase of cell cycle.[13] In this study, a positive correlation was observed between multi-shoot susceptibility to *Agrobacterium* and the proportion of cells in G1 phase in the explants at the time of initiation of the co-cultivation period. Cell cycle is also suggested to play a critical role in the interaction between plant cells and *Agrobacterium*. In particular, histones (H2A, H2B, H3 and H4) that accumulate during the S phase of cell cycle may play a role in *Agrobacterium*-mediated transformation. The study in *Arabidopsis* revealed that the meristematic region of the root that showed highest histone H2A-1 gene activity was also the most transformation-competent. There is a remarkable correlation between histone H2A-1 gene activity and susceptibility to *Agrobacterium* transformation.[35]

Factors that, in the present study, affected the efficiency of *Agrobacterium*-mediated transient expression of GUS included *Agrobacterium* strain, corn genotype, the stage of corn tissues infected, composition of medium for co-cultivation, co-cultivation temperature and surfactant treatment. Vega et al. [7] found that some of the same factors including corn genotype, transformation vector, components of the media and explant stage affected the success of immature embryo transformation in corn.

We found that the agropine strains of *Agrobacterium* (EHA101 and AGL1) were eightfold more effective in infecting corn shoot-tip and multi-shoot tissues than an octopine-type strain (LBA4404). In contrast, Ritchie et al. [36] reported that agropine-type strains have the highest efficiencies of GUS activity in the mesocotyl tissues, but lower efficiency than the nopaline strain in the shoot meristem segment. However, we have not observed an apparent enhancement of *Agrobacterium* infection in shoot meristem region by nopaline strain (data not shown).

In the previous report,[13] we demonstrated that the supplementation of proline in multi-shoot inducing medium obviously gained the differentiation of callus. Together with the new finding that a moderate addition of proline in co-culture medium stimulated the growth of *A. tumefaciens* in the present study (data not shown) and the subsequent increment of T-DNA delivering efficacy, we believe that the enhancement of proline to the development of both plant cell and *A. tumefaciens* results in the improvement of corn transformation. Meanwhile, we found that when the surface of explants was treated with a low dose of surfactant SilwetL-70 (0.05%), the transformation efficacy was improved perhaps due to the surface-tension-free cells favouring the *A. tumefaciens* attachment, similar to the response in *Arabidopsis* transformation.[37]

Most of the transient GUS expression obtained in the present study was not maintained as stable transformants (data not shown), which could be the main reason that caused the lower frequency of transgenic plants compared to the reports of Ishida et al. [18] and Frame et al. [6]. Therefore, our ongoing emphasis will be placed on increasing the frequency of stable T-DNA integration.

## Conclusions

Our results showed that *A. tumefaciens* strains, corn genotypes, corn tissue culture stages, medium compositions, co-cultivation temperature and surfactant treatment significantly influence the GUS gene transfer into corn tissue. The frequency of transformants was approximately 2% based on the number of multi-shoot explants that were co-cultivated with *Agrobacterium*.
